# Research Participation among Community Dwelling Dementia Caregivers: Reflections and Suggestions

**DOI:** 10.1093/geroni/igab046.2926

**Published:** 2021-12-17

**Authors:** Kiana Cruz, Sama Joshi, Taeyoung Park, M Carrington Reid, Keela Herr, Karl Pillemer, Catherine Riffin

**Affiliations:** 1 Weill Cornell Medical College, New York, New York, United States; 2 Cornell University, Ithaca, New York, United States; 3 University of Iowa, Iowa City, Iowa, United States; 4 Weill Cornell Medical College, London, England, United Kingdom

## Abstract

Clinical trials for dementia caregivers have suffered from small sample sizes that lack adequate power to detect treatment benefits. Addressing these methodological shortcomings is contingent upon successful recruitment and enrollment of caregiver participants, but major barriers impede their participation in research. This presentation describes the lessons learned from recruiting and enrolling dementia caregivers into a pilot randomized controlled trial designed to help caregivers recognize and communicate about pain in dementia care recipients. Using Bronfenbrenner’s ecological model, we organize our discussion of challenges and opportunities into three levels: community (ecosystem), institution (microsystem), and individual. A key challenge at the community level was gatekeeping by organization leaders, including those from support groups, senior centers, and congregate living facilities. At the institutional-level, challenges included an absence of administrative mechanisms for identifying caregivers and a lack of caregiver research expertise on the Institutional Review Board. At the individual-level, challenges included time constraints and varying motivations for participating in research. Strategies for overcoming these challenges spanned the three levels and included establishing trust and rapport with various constituencies; adapting our recruitment approaches to meet the specific motivations of prospective participants; and refining recruitment scripts to allow for greater personalization. Employing these strategies, which can be generalized to recruit other hard-to-reach populations, helped to overcome recruitment challenges and expedite enrollment of caregivers from a diverse range of sociodemographic backgrounds. Further improvement will require coordinated changes at the institutional and community levels, including the development of central research registries and administrative mechanisms for identifying caregivers.

